# Neill on the Nerves

**Published:** 1846-01

**Authors:** 


NEILL ON THE NERVES.

We noticed, some time ago, a work on the Arteries, by Dr. Neill. The volume before us is of the same character, being designed to give to students a general idea of the anatomy of the nerves. As in the former production, the task has bɘen executed with fidelity and skill, and in the plates delineating the origin and course of the nerves the student will find a clear and beautiful exhibition of this portion of anatomy. We heartily recommend the work to all students of medicine. Y.

